# Experimental Identification of Smartphones Using Fingerprints of Built-In Micro-Electro Mechanical Systems (MEMS)

**DOI:** 10.3390/s16060818

**Published:** 2016-06-03

**Authors:** Gianmarco Baldini, Gary Steri, Franc Dimc, Raimondo Giuliani, Roman Kamnik

**Affiliations:** 1 European Commission, Joint Research Centre, Ispra 21027, Italy; gary.steri@jrc.ec.europa.eu (G.S.); raimondo.giuliani@jrc.ec.europa.eu (R.G.); 2 Faculty of Maritime Studies and Transport, University of Ljubljana, Portorož 6320, Slovenia; franc.dimc@fpp.uni-lj.si; 3 Faculty of Electrical Engineering, University of Ljubljana, Ljubljana SI 1000, Slovenia; roman.kamnik@fe.uni-lj.si

**Keywords:** MEMS, fingerprinting, accelerometers, gyroscopes, counterfeit, smartphone

## Abstract

The correct identification of smartphones has various applications in the field of security or the fight against counterfeiting. As the level of sophistication in counterfeit electronics increases, detection procedures must become more accurate but also not destructive for the smartphone under testing. Some components of the smartphone are more likely to reveal their authenticity even without a physical inspection, since they are characterized by hardware fingerprints detectable by simply examining the data they provide. This is the case of MEMS (Micro Electro-Mechanical Systems) components like accelerometers and gyroscopes, where tiny differences and imprecisions in the manufacturing process determine unique patterns in the data output. In this paper, we present the experimental evaluation of the identification of smartphones through their built-in MEMS components. In our study, three different phones of the same model are subject to repeatable movements (composing a repeatable scenario) using an high precision robotic arm. The measurements from MEMS for each repeatable scenario are collected and analyzed. The identification algorithm is based on the extraction of the statistical features of the collected data for each scenario. The features are used in a support vector machine (SVM) classifier to identify the smartphone. The results of the evaluation are presented for different combinations of features and Inertial Measurement Unit (IMU) outputs, which show that detection accuracy of higher than 90% is achievable.

## 1. Introduction

Correct identification of electronic devices including smartphones is an important function in various applications including the fight against counterfeiting. Counterfeit goods have reached a level of precision and similarity to the original ones that their detection cannot be limited anymore to a visual inspection of the external appearance. Nowadays, counterfeiters of electronic devices have knowledge, expertise and equipment comparable to ones owned or managed by the original component manufacturers (OCMs). Counterfeit parts of an electronic device, including the integrated circuits (ICs), are not only clones but also recycled, overproduced or (different) remarked components as described in a recent study [1]. This means that, in many cases, they are still components coming from the production line of the OCM or from its suppliers but with lower quality, perhaps posing problems of reliability and security of the devices, as already reported even in the automotive, aviation and military industry.

Under these circumstances, counterfeit devices result in a surprisingly high level of similarity to the original ones, so that it is difficult to decide about their authenticity with visual inspection of the external case only or with inspection of internal components. For this reason, different inspection techniques have been developed and employed. Low-power visual inspection (LPVI) with microscopes or magnification lamps, X-ray imaging and fluorescence, Scanning Acoustic Microscopy (SAM) and Scanning Electron Microscopy (SEM), and parametric and structural tests are only a few examples of the physical and electrical inspections that can be applied. However, most of these techniques require complex and expensive equipment, specialized technicians, and a considerable amount of time to execute. In addition, many techniques are destructive as they require the opening of the smartphone or the removal of specific components. The application of the SEM technique for the inspection of an IC’s die, for example, requires the decapsulation of the component, while the microblast analysis requires the removal of the markings on the surface of the inspected part in an attempt to find previously printed markings.

In light of the above considerations, in this paper, we investigate a technique for the identification of smartphones that does not require disassembling the smartphone and that can be applied by a simple process and test bed equipment. The technique is based on the consideration that modern smartphones have built-in Micro-Electro Mechanical Systems (MEMS) sensors such as accelerometers and gyroscopes, which are uniquely characterized by unreproducible hardware fingerprints, allowing even for distinguishing between two original devices (*i.e.*, different serial numbers) of the same model [2]. An important element of this technique is that these fingerprints can be read by simply analyzing the values processed by the MEMS components and collected in the smartphone memory without requiring the disassembling of the smartphone. A practical deployment of this technique could exploit the collection of MEMS digital output during the device hardware reliability tests as defined by the Cellular Telephone Industries Association (CTIA) (now The Wireless Association) [3], where smartphones are subject to rotations or other motion scenarios (e.g., TumbleBarrel Test).

The MEMS components present in a smartphone have been investigated for a variety of applications by the research community. In [4], the authors have investigated the potential of the MEMS in the smartphones to automatically detect the fall of a person carrying a smartphone. This is a useful application for healthcare, because falls are the foremost source of injuries and hospitalization for seniors. The results of the experimental testbed in [4] show that the accelerometer-based techniques to identify the falls depends strongly on the fall pattern. This is valid input for the study presented in this paper, which supports the concept that the movement used for fingerprinting the smartphone must be well defined and reproducible. Another example of the use of MEMS components in a smartphone is presented in [5], where the authors have investigated whether the phone position can be detected with high accuracy by analyzing the movement, orientation and rotation changes. It is shown that while utilizing only motion information, they can achieve accuracies around 70%, this ratio increases up to 85% by utilizing information also from orientation and rotation changes. Other examples of human movement tracking and assessment using MEMS (in particular, accelerometers and gyroscopes) are in [6,7], where the same authors perform a three-dimensional reconstruction of the movement instead of analyzing sensors’ output as a waveform. These results prompted us to define a movement pattern with a complete set of movements and rotations in 3D space.

In addition to the application of fighting against counterfeiting, the identification of the smartphone also has other applications in the fields of security or privacy. The authentication of the smartphone on the basis of intrinsic features created during the manufacturing process can be used to support security applications. For example, the MEMS based identification can support a more conventional identification based on cryptographic means (e.g., Public Key Infrastructure certificates). On the other hand, the identification of smartphones and their owners is an important element to take into consideration in a privacy risks analysis because users could be traced on the basis of their smartphone even if anonymization techniques are adopted. For example, an application could extract content data including MEMS fingerprints from a smartphone to uniquely identify the smartphone even if the content data has been anonymized by removing the identifier of the serial number of the smartphone. Obviously, this privacy threat is possible only if the unique MEMS fingerprints have been already collected and processed previously.

Exploiting these characteristics of MEMS components, our contribution in this paper is to evaluate the feasibility of fingerprinting the smartphones with their built-in MEMS through a procedure that can be deterministically reproduced in different forensics labs without the need of complex equipment and infrastructures. The application scenario foresees the creation of an archive of devices’ fingerprints obtained with a standard sequence of movements (composing a repeatable scenario) applied to the device using a computer-activated mechanical platform, and, during the inspection phase, the extraction of the inspected device’s fingerprint performing exactly the same movement and solicitation on the device, in order to compare the extracted fingerprint to the ones contained in the archive, minimizing errors due to external factors or differences in the movement. We extract the statistical features from the collected MEMS digital output and we use Support Vector Model (SVM) classification for the identification of the phones. Before building such a reference library with fingerprints of many different phones, it is important to evaluate and identify the statistical features or algorithms that provide the best identification accuracy. This step is the main objective of this paper. We focus on the experimental evaluation and selection of the statistical features on a limited set of phones of the same model, which only differ for the serial numbers. We conduct an extensive evaluation of the different components of the MEMS (accelerometers, gyroscopes), statistical features and different kernel functions for the SVM binary classification algorithm. In comparison to the results provided in literature as described in Section 2, we use a more extensive set of statistical features, which also include entropy based features. We believe that this experimental evaluation is important in order to evaluate the feasibility of phone identification through MEMS, and it is useful for researchers in this domain.

The remainder of the paper is organized as follows: Section 2 provides the state-of-the-art MEMS sensor fingerprinting, counterfeit electronics, and smart-device detection techniques. Section 3 provides the overall methodology for the fingerprinting data collection, analysis and comparison. Section 4 shows the results of our tests, and Section 5 provides the final considerations and future work.

## 2. Related Work

### 2.1. Motion Sensor Fingerprinting

Device identification based on MEMS sensor fingerprinting, and, in particular, on accelerometers, has been presented mainly in [2,8]. Both studies face the problem of device and user identification made by cloud applications remotely accessing the data sensed by the on-board accelerometer.

In [2], which actually also analyzes stand-alone sensors, fingerprints are extracted while the device is vibrating. The analyses of *traces* of 2 s of duration, leads to the extraction of a set of features to which supervised learning is applied and verification of whether it is possible to distinguish between different accelerometers. The results actually show an overall percentage of precision and recall above 87% when the classifier is trained with at least 30 s of vibration. However, among these results, there are some distinctions related to the type of device (stand-alone sensors or sensors built-in a smartphone or tablet) and to the surface on which the device is lying, as well as the presence of a casing on the device. All these variables are analyzed, and the biggest influence comes from the casing that, in some circumstances, reduces precision and recall around 60%, while on the different surfaces, it is still possible to reach almost 80%. Moreover, the overall best results are for standalone chips, *i.e.*, accelerometers mounted on a breakout board and connected to an Arduino board for data collection. This case has clear limitations in real scenarios where, as in our case, it is necessary to operate on the device *as it is*, not on a single component that also cannot be easily removed for testing.

In [8], where the fingerprinting of a loudspeaker and microphone is also proposed, accelerometer data are collected when the device is lying still on a surface with the *z*-axis perpendicular to it, first facing up and then facing down. In this case, no movements are required, but the gravity force alone is used to elicit the reaction of the sensor and extract its fingerprint. Sensitivity and offset of the sensors are taken as reference parameters in order to calculate the distance between data and identify the devices. The lab experiment with 17 devices yielded percentages of correct identification above 85% for some scaling factor between the two parameters, and the large scale experiment conducted on more than three thousand devices sending their data through an on-line page reached only 15%, increasing to 58% using the User-Agent string. However, the use of a software identifier would invalidate all the assumptions for the need of hardware identifiers in our and in other scenarios.

The problem of smartphone’s motion sensor fingerprinting also inspired some work on mitigation techniques. In [9], authors first developed their own fingerprinting mechanism using both the accelerometer and gyroscope of the smartphone, collecting the data with a stationary device with and without audio stimulation. Combined results from both lab and real-world experiments featured an F-score of 96%, showing also that the gyroscope gives better results under (inaudible, 20 KHz) audio stimulation compared to the accelerometer. On the basis of these results, the authors propose some countermeasures in order to make sensor fingerprinting more difficult and then user identification, showing an F-score reduction of up to 30% after sensor calibration (much less for the gyroscope, which is more difficult to calibrate), and of up to 40% and 80% applying data obfuscation, respectively, with and without injection of new data samples. However, data obfuscation and fingerprint *scrubbing*, in general, can result in heavy affection of application’s functionalities.

### 2.2. Counterfeit Smartphone Detection

Authors in [10] propose a counterfeit mobile phone identification based on Visual Cryptography Scheme (VCS). The idea is to protect the unique serial number of the device using VCS, so that one share is stored in the phone and the other one resides in the online verification service of the manufacturer. However, authors do not consider the possibility for a counterfeiter to dump the binary image stored in the phone, which would still result in a successful verification.

Other anti-counterfeiting techniques, applicable not only to mobile phones but potentially to any object, are based on radio frequency fingerprinting. In [11], Radio Frequency Identifiers (RFID) tags are made physically unique and are difficult to replicate using RF certificates of authenticity (RF-CoAs). Even though these methods could represent valid countermeasures, they have to be applied to new devices, and they cannot be implemented on the current ones. To the best of our knowledge, there are no approaches that exploit MEMS fingerprints to detect counterfeit smart devices.

The future of anti-counterfeiting techniques is still based on fingerprint detection, but the fingerprints will be consciously inserted during the manufacturing process so that they can be verified in order to identify the authenticity of a product. This is the case of the Physical Unclonable Functions (PUFs) applied to silicon chips [12], which determine speed differences in response to electrical signals. When several pairs of challenge inputs are sent to the chip through different paths, the results of which signal in each pair first reached a target latch will give a string of bits identifying the chip. In a similar way but in a different context, researchers in [13] have developed a technique to apply nanoscale fingerprints in Polyethylene terephthalate (PET) films in order to authenticate different objects by randomly inserting silver nanowires whose disposition results in non-repeatable patterns.

## 3. Methodology for Data Acquisition and Processing

**Figure 1:**
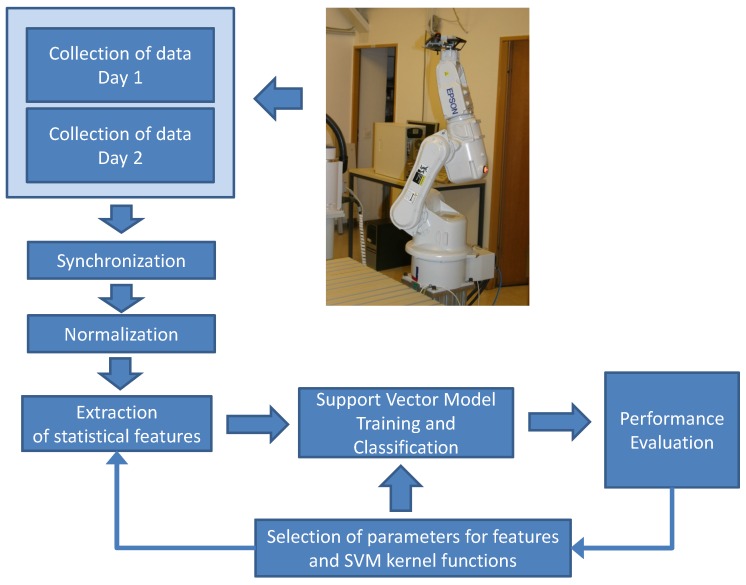
Workflow of the adopted methodology.

The overall methodology flow used in the paper for the collection of data, processing and analysis is shown in Figure 1.

The first step is the collection of MEMS data. Observing the other studies on motion sensor fingerprinting presented in Section 2, we noticed that the level of precision is influenced by several factors, such as the duration and type of stimulus (if any) applied to sensors (vibration or audio stimulation), position and wrapping of the sensors (stand-alone chip or chip embedded in a device with possible casing), estimation of offset and sensitivity of the sensors. To avoid all of these problems and improve the accuracy of the classification, we relied on a mechanical platform on which the device is always placed exactly in the same positions, and it is always subject to the same controllable and repeatable movements.

**Figure 2:**
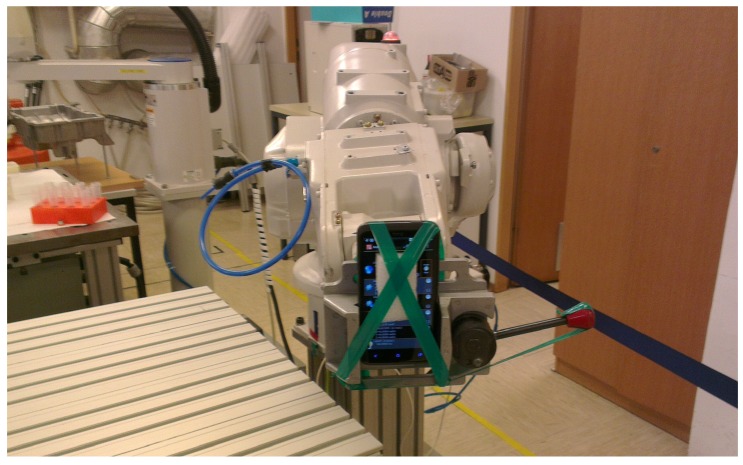
Robotic Arm used in the scenario with one of the Phones attached.

The scenario for collecting the MEMS data from the phones is following: an EPSON (Nagano, Japan) PS3 robotic arm with six Degrees of Freedom (DOF) has been used to manipulate each phone in a specific motion scenario with known accelerations. The scenario consists of a lateral movement of 120 degrees in three steps of 40 degrees each on the *x*-*y* plane. For each step, the mechanical arm raises from 0 degree elevation to 30 degrees. When the robotic arm reaches the final position at 90 degrees elevation and 120 degrees on the *x*-*y* plane, it reverts to the initial position. These sets of movements ensure that all the MEMS components are stimulated to generate significant output values for the analysis. A picture of the phone attached to a robotic arm is shown in Figure 2. Before starting each scenario, the smartphones were kept still for 20 min to ensure that the initial settings of the MEMS were the same, and the previous scenario did not influence the results of the subsequent scenario. While we agree that an EPSON PS3 robotic arm could be expensive equipment for practical purposes, in this paper, we wanted to ensure the repeatability of the motion pattern. Results from the literature as described in Section 2 shows that a good level of accuracy can also be obtained with less expensive equipment. A comparison of different platforms for the generation of the motion scenarios is future research, and it is not addressed in this paper.

The data of the built-in MEMS was acquired on the phone using the Androsensor application configured to collect the data of accelerometers and gyroscopes at the rate of 10 Hz.

The measurement scenario was repeated 96 times to provide enough data for the statistical analysis for all three phones used in the experiment in four different sessions in four days to evaluate the accuracy and robustness of the fingerprinting algorithm for a collection of samples. The first two sessions were executed with a time gap of a few days. After four months, another two sessions were executed again with a time gap of a few days. In other words, it is important to evaluate the algorithm both for (a) distinguishing two smartphones of the same model with different serial numbers and (b) for providing the same identification decision (e.g., the same smartphone) even if the collected samples are on different days when environment conditions like ambient temperature or humidity could be different. Both evaluation criteria are important, and they are measured for different MEMS components and parameters in Section 4.

**Figure 3:**
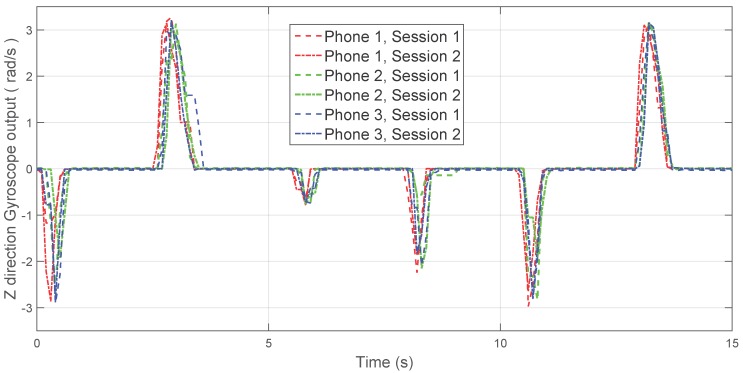
*z*-output from the Gyroscopes as received from the normal axis to the frontal plane of the phones.

The second step in data processing is the synchronization and normalization of the collected data for each scenario, which is needed to ensure that the accuracy of the algorithm is not biased by potential different configurations in the phones. An example of the resulting normalized *z*-axis outputs of the Gyroscope, *i.e.*, angular rate results acquired around the normal axis to the frontal plane of the phone, is shown in Figure 3.

The third step is to apply statistical analysis to the synchronized and normalized samples. To each scenario, we applied the following statistical features in the time domain and on the absolute value $S_{i}$ of the sensor data, where *i* is the sample identifier. The entropy features are derived from the MATLAB wavelet toolbox.

(1) $$variance\left(\sigma \right)=\frac{1}{N-1}\sum_{i=1}^{N}{\left(S_{i}-\mu \right)}^{2},$$

(2) $$Skewness=\frac{1}{\sigma^{3}}\sum_{i=1}^{N}\left(S_{i}^{3}-\mu^{3}\right),$$

(3) $$Kurtosis=\frac{1}{\sigma^{4}}\sum_{i=1}^{N}\left(S_{i}^{4}-\mu^{4}\right),$$

(4) $$ShannonEntropy=-\sum_{i=1}^{N}\left(S_{i}^{2}\right)*Ln\left(S_{i}^{2}\right),$$

(5) $$LogEnergyEntropy=\sum_{i=1}^{N}Ln(S_{i}^{2}),$$

(6) $$ThresholdEntropy=1\;if\;|S_{i}|>P_{Thr}\;and\;0\;elsewhere.$$

(7) $$SureEntropy=N-\;(i\;if\;|S_{i}\left|<=P_{Thr}\right)\;+\;\sum_{i=1}^{N}min(S_{i}^{2},{P_{Thr}}^{2}),$$

(8) $$NormEntropy=\sum_{i=1}^{N}|S_{i}{|}^{P_{Thr}}.$$

These statistical features were adopted because they are widely used in device identification and fingerprinting. For example, a similar set of statistical features have been used by [14] to classify and identify RFID tags. Variance, skewness and kurtosis were also used by [15] to fingerprint 16-bit Peripheral Interface Controller (PIC) micro-controllers.

The calculated statistical features were applied to the MEMS digital output produced in the 96 repetitions to generate a matrix of 96 rows for the eight features for each smartphone and each scenario. This means that we formed 3 × 2 matrices of dimension 96 × 8 to which we applied supervised learning. We submitted the generated matrices to an SVM classifier with different kernel functions. From the 96 repetitions, 72 repetitions were used for training and 24 repetitions for the holdout validation. The metric used for our results is the identification accuracy, which is calculated as $Accuracy=\frac{T_{p}+T_{n}}{TotalPopulation}$, where $T_{p}$ is the number of True positives and $T_{n}$ is the number of True negatives. We used SVM as a binary-classifier to compare the digital output (*i.e.*, generated feature matrix) of each smartphone with another smartphone or for different scenarios.

The following SVM kernel functions are used:

1. Gaussian Radial Basis Function (RBF) kernel with different values of the scaling factor *σ*.
2. Multilayer Perceptron (MLP) kernel, which is a feed-forward artificial neural network model that maps sets of input data onto a set of appropriate outputs. We used a scale which goes from −1 to 1.
3. A linear kernel.
4. Quadratic kernel.
5. Polynomial kernel (with different orders).

The SVM was used with the Sequential Minimal Optimization (SMO) method and we specified the fraction of variables (we denoted it as kkt level in the rest of the paper) allowed to violate the Karush–Kuhn–Tucker (KKT) conditions for the SMO training method.

The theory of SVM is well known and the reader can refer to [16] for further details.

## 4. Experimental Results

In this section, we show the results of the experimental tests to evaluate how different parameters and different SVM kernel functions can be used to distinguish the different phones. As described in Section 3, two sets of measurements were taken on different days to evaluate the robustness of the algorithm against time.

**Figure 4:**
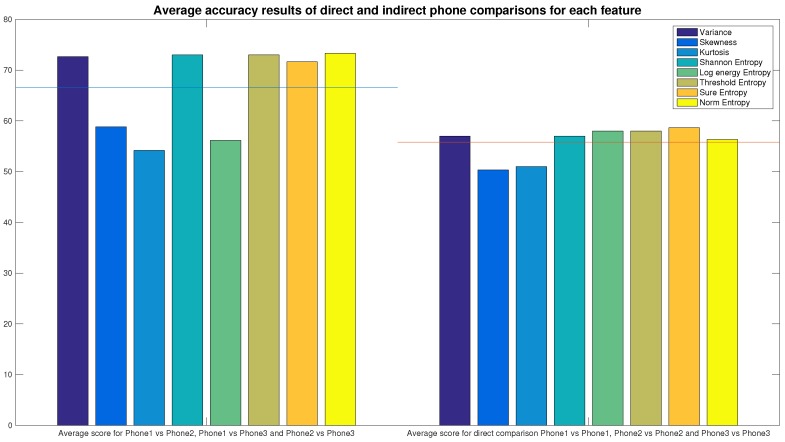
Average scores of comparison of each phone against each other and against itself for different features: horizontal lines show the average values for all the features (66.6 and 55.7, respectively).

In the following results, we evaluate the performance of the Accelerometer’s *X* output. In Section 4.4, we evaluate the performance for different components of the MEMS and other axes of the accelerometer. Since some features work better than others, in order to decide which features are the best, we need to compare them directly. To do this, we calculate the average score of all the comparisons of each phone against the others, as shown in Figure 4. The results were obtained by using the SVM with a Gaussian Radial Basis Function (RBF), a *σ* parameter equal to 0.5 and a kkt level equal to 0.1. This was just a starting setting as, in the next sections, we will optimize the parameters both for statistical features and kernel functions.

### 4.1. Features Optimization

**Figure 5:**
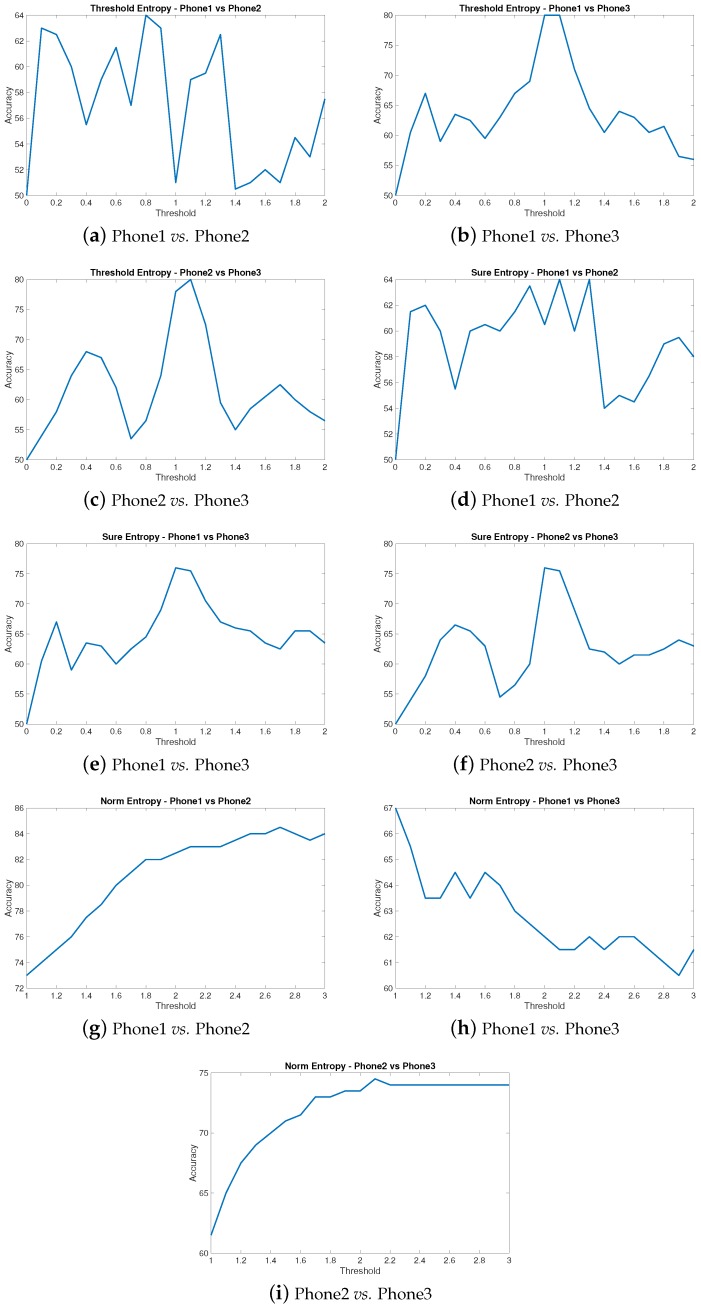
Threshold $P_{Thr}$ variation and accuracy results for *Threshold Entropy*, *Sure Entropy* and *Norm Entropy* features. The values that give the best average accuracies are, respectively, 1.1 (average 73.0), 1.1 (average 71.6) and 2.5 (average 73.3).

Some of the statistical features have parameters that can lead to different accuracy results. In particular, *Threshold Entropy*, *Sure Entropy* and *Norm Entropy* require the setting of a *threshold* value. Different values of the threshold can provide different values of the accuracy. We experimentally identified the best values of the threshold. The accuracy scores shown in Figure 4 were calculated with the threshold value giving the best average accuracy for each comparison. Graphs showing the effect of different values of the threshold $P_{Thr}$ on the three entropy features above are depicted in Figure 5. Note that, for the Norm Entropy, the threshold has to start from 1. As in the previous results, we used the RBF kernel function with a sigma parameter equal to 0.5 and a kkt level of 0.1. We note that some values of the threshold are difficult to select for some entropy statistical features (e.g., norm entropy) and some minor variations in the environment or the MEMS could change the optimum threshold values. This can become a concern for applications where a high and reproducible accuracy is requested (e.g., security). In these cases, an application designer may prefer to select other entropy statistical features.

### 4.2. Optimizing the Training Algorithm

Similarly to the features, SVM kernel functions can be optimized adjusting some settings and parameters. For example, for the RBF kernel function, we can evaluate the effects of the *σ* scale factor. We found out that values of sigma close to 0 give the lowest scores, while after 1 (standard value), the average approaches the maximum (67.1), which is reached when sigma equals 1.6 (we run tests with sigma up to 10).

In the same way, we analyze the effect of the *order* parameter in the polynomial kernel function. The order of the polynomial does not influence the average accuracy much in the different phone comparisons (Phone1 *vs.* Phone2, Phone1 *vs.* Phone3 and Phone2 *vs.* Phone3). However, the order 3, already used in the previous calculations, gives the best result (66.7).

Using these parameters, we can now compare all the kernel functions in order to choose the one giving the best accuracy. The best average accuracy (67.1%) is obtained using the RBF kernel function with the optimized value of sigma (1.6). However, the linear kernel function reaches almost the same average (66.7 %).

### 4.3. Features Combination

Using the best algorithm configuration found in the previous Section, we now combine the features in order to improve the accuracy of the identification. We saw already in Figure 4 that Variance and entropies (except *Log Energy Entropy*) featured the best average accuracy results. Starting from groups of two features up to the use of all of them together, we show which combination gives the best results.

#### 4.3.1. Combination in Groups of Two, Three, Four and Five Features

**Figure 6:**
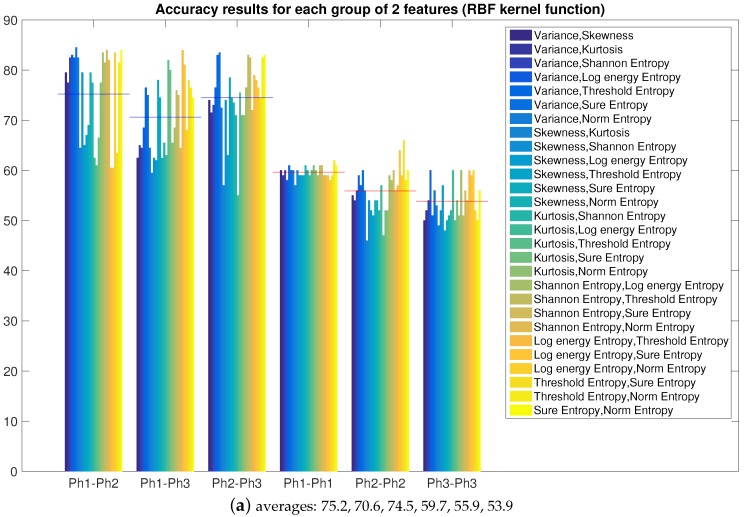

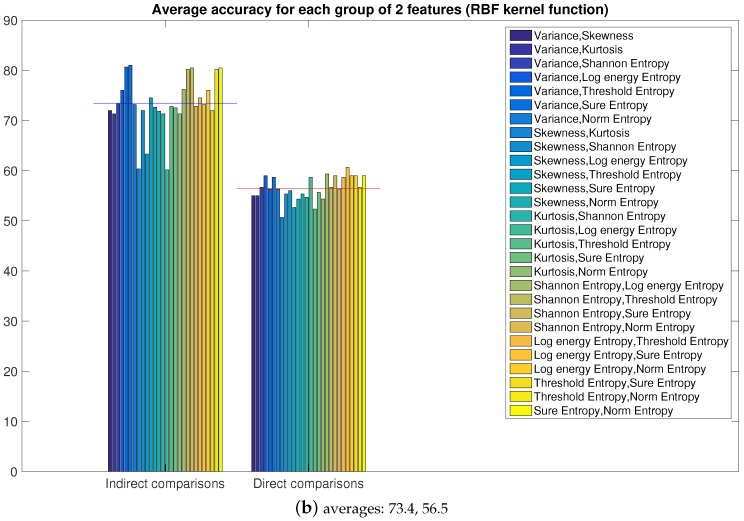
Accuracy and average accuracy for groups of two features (Radial Basis Function (RBF) kernel function, sigma 1.6).

Figure 6 shows the 28 combinations of two featured as grouped by comparison (Phone1 *vs.* Phone2, *etc.*) both for single and average results. It is already possible to see that when Variance and entropies are combined together, the accuracy is higher.

**Figure 7:**
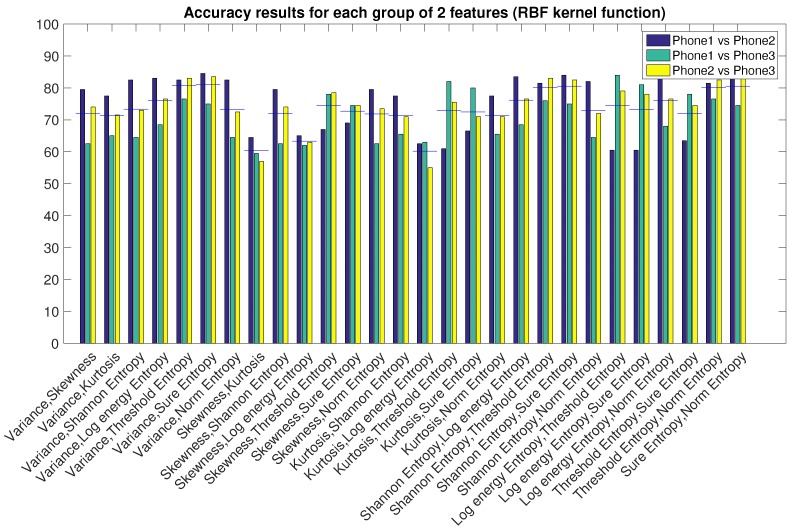
Features combination in groups of two for indirect comparisons. Best group: *Variance, Sure Entropy*, Average accuracy of 81%.

In order to be sure about the best combination, we show the same results grouped by pairs of features, as done in Figure 7. The best result (we now show only *Indirect comparisons, i.e., Phone1 vs. 2, 1 vs. 3 and 2 vs. 3*) is obtained with the pair *Variance, Sure Entropy*, with an average accuracy for three comparisons of 81%, which is better than the result obtained for single features.

Following the same approach, we now combine features in groups of three (56 combinations), where the best result is given by the group *Variance, Log Energy Entropy, Threshold Entropy*, which scores an average accuracy of 83.3% in the comparison of the three phones. Here, features are numbered following the order presented in Section 3. Again, the increase of the number of features improves the accuracy.

Groups of four features are the most numerous (70 combinations), and they allow for improving the accuracy a bit more. The best group is *Log Energy Entropy, Threshold Entropy, Sure Entropy, Norm Entropy*, with an average result of 83.6%. The relevance of the entropy statistical features for the identification of the smartphone on the basis of the built-in MEMS confirms the initial findings of [8], which noted significant variations in the Shannon Entropy of the accelerometers in the *z*-axis, but it did not explore it further. Indeed, the authors in [8] pose a question in the Conclusions section on how much entropy can be extracted overall from the MEMS sensors and how it can be used for identification. This paper addresses and provides results for this question. Our findings are based on a more extensive set of entropy statistical features, and we compared in a more detailed way the performance of the accuracy. We also note that the application of entropy statistical features was not used in other recent papers identified in Section 2. For example, the authors in [2] used more conventional statistical features (e.g., variance, skewness and kurtosis), which are also included here, but they do not use any entropy features.

With five features per group, the number of combinations start decreasing (56), but the accuracy increases to 84% with the group *Shannon Entropy, Log Energy Entropy, Threshold Entropy, Sure Entropy, Norm Entropy*.

#### 4.3.2. Combination in Groups of Six, Seven and Eight Features

When grouping six features (28 groups), the accuracy starts to decrease. The best score, given by the group *Variance, Kurtosis, Log Energy Entropy, Threshold Entropy, Sure Entropy, Norm Entropy*, is 83.3%.

In the same way, grouping by seven (eight combinations) reduces the accuracy to 82.5% (features: *Variance, Kurtosis, Shannon Entropy, Log Energy Entropy, Threshold Entropy, Sure Entropy, Norm Entropy*.

Finally, when combining all eight features together, the average result for indirect comparisons drops to 80.3% (81% for Phone1 *vs.* Phone2, 78.5% for Phone1 *vs.* Phone3 and 81.5% for Phone2 *vs.* Phone3).

### 4.4. Analysis of MEMS Components

In the previous results, we have used the collected and processed data related to the Accelerometer’s *X* output. However, different sensors and outputs can give better results, since the movement of the robotic arm can have different effects depending on how accelerations and rotations are performed. For this reason, in this section, we compare the performances of each axes of both Accelerometer and Gyroscope, in order to see which data allow for the best accuracy.

**Figure 8:**
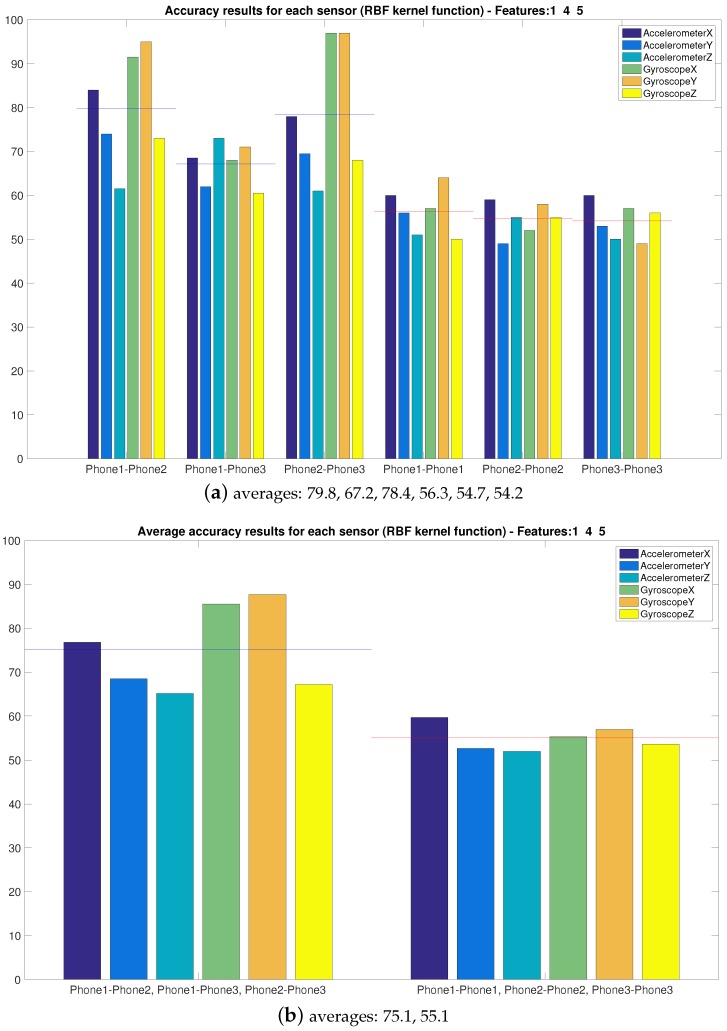
Accuracy for features one, four and five for all of the Microelectromechanical (MEMS) components.

We first do a comparison of the MEMS components using a group of three features (*i.e.*, Variance (1), Shannon Entropy (4) and Log Energy Entropy (5)) that do not require the adjustment of parameters (e.g., $P_{Thr}$ of the other entropy features). Results are shown in Figure 8, and we can see that the accuracy obtained from *x* and *y* outputs of the Gyroscopes is much higher than from the Accelerometers’ outputs.

Now, as is done for the Accelerometer’s output *X*, we optimize the threshold $P_{Thr}$ for the three entropy features (six, seven and eight). The results, including all the axes of all the sensors, are summarized in Table 1.

**Figure 9:**
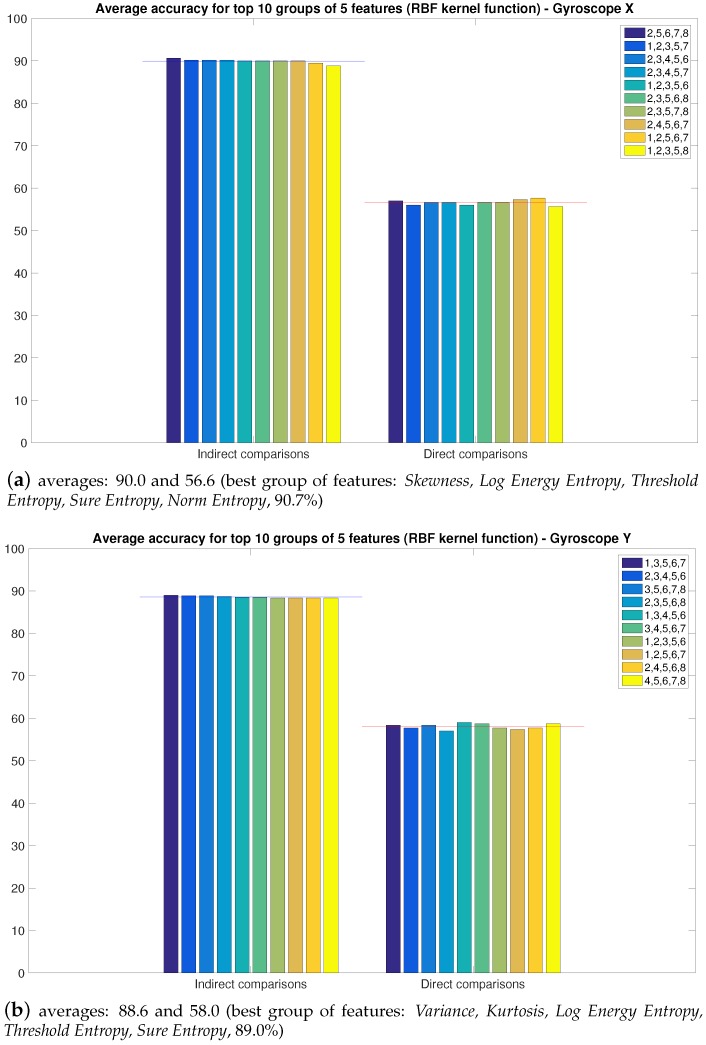
Accuracy for top 10 groups of five features for Gyroscope *x* and *y* outputs.

Using these settings, we can finally calculate the accuracy for the *x* and *y* outputs of the Gyroscope with groups of five features, and we show the average results for the top 10 combinations in Figure 9 (by top 10, we mean the 10 highest scores in the average indirect comparison).

These results show that it is possible to distinguish different smartphones of the same model with high accuracy, while the algorithm almost provides correctly random choice accuracy for the observables taken on different days.

In order to ensure the generalizability of the method, we have repeated the measurements and the collection of the digital output from the MEMS at a distance of four months from the initial measurements that have been used to generate the results above. We have used the same test set-up (*i.e.*, the same platform and the same motion scenarios) and the same mobile phones used in the previous experiment for two different days.

The results are shown in Table 2 for the Gyroscope X and in Table 3 for Gyroscope Y and for different sets of statical features extracted from the top groups presented in Figure 9.

We observe a degradation in the accuracy for the gyroscope X (around 80% accuracy instead of around 90% accuracy four months before), but the values of the accuracy for the gyroscope Y are quite similar to the ones obtained four months before.

Now that the best sets of features have been selected, we examine more in detail the classification accuracy on the basis of Receiver Operating Characteristic (ROC) and Equal Error Rate (EER). While the accuracy, which has been used in the previous graphs, is based on the True Positives (TP) and True Negatives (TN), these additional metrics give an estimate of the probability of False Positives (FP) and False Negatives (FN) as well. A FP represents the result, which indicates that a given condition has been fulfilled, when it actually has not been fulfilled. A FN is a test result, which indicates that a condition failed, while it actually was successful.

The Receiver Operating Characteristic (ROC) is a graphical plot that illustrates the performance of a binary classifier system (SVM in this paper) as its discrimination threshold is varied. The ROC curve is created by plotting the rate of TP (TPR) against the rate of FP (FPR) at various threshold settings across the interval [0,1].

In relation to the ROC, another common metric used to evaluate a classification system is the Equal Error Rate (EER), which corresponds to the point on the ROC curve where the rate of FN (equal to 1—TPR) and FPR are equal. This metric is frequently used as a summary statistic to compare the performance of various classification systems. In general, a lower EER indicates a better system classification performance.

**Figure 10:**
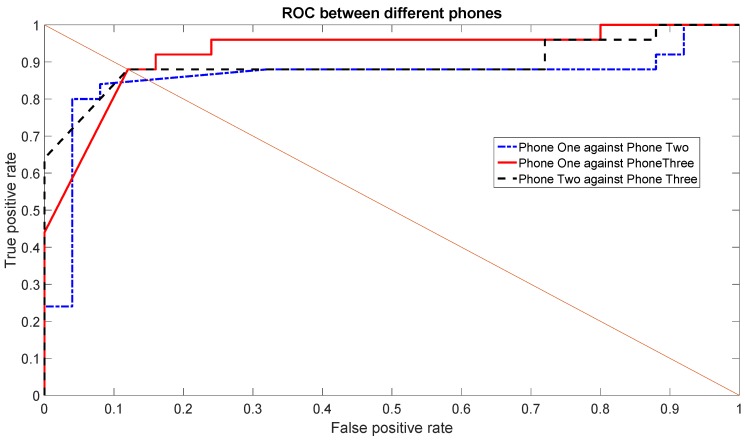
Receiver Operating Characteristic (ROC) among different phones using the first scenario for Gyroscope X for a single day’s data in the first set of measurements.

**Figure 11:**
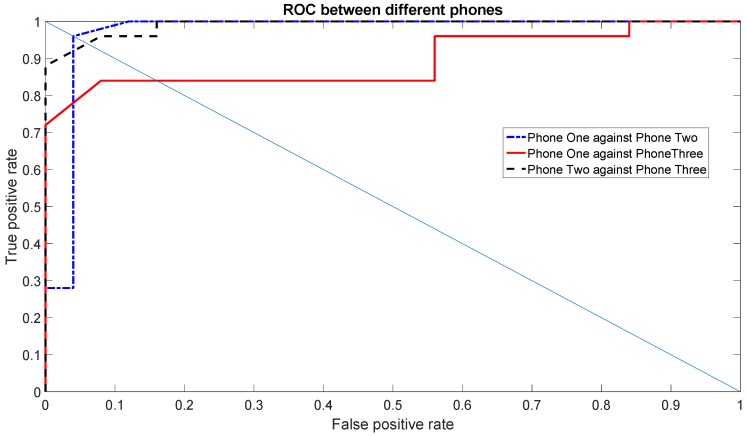
ROC among different phones using the first scenario for Gyroscope Y for a single day’s data in the first set of measurements.

Figure 10 shows the ROC using the Gyroscope X for the set of features (1, 2, 3, 5, 7), which was one of the top sets of features. Figure 11 shows the ROC using the Gyroscope Y for the set of features (1, 3, 5, 6, 7) using the data of one day. Both ROC graphs show the comparison between the different Phones: One against Three, Two against Three and One against Two. The straight line is used to show how the EER is calculated. The resulting values of the EER are shown in Table 4. The values of the EERs show that Gyroscope Y usually has a better performance than Gyroscope X as also shown by the data taken in the second round of measurements (see analysis below).

**Figure 12:**
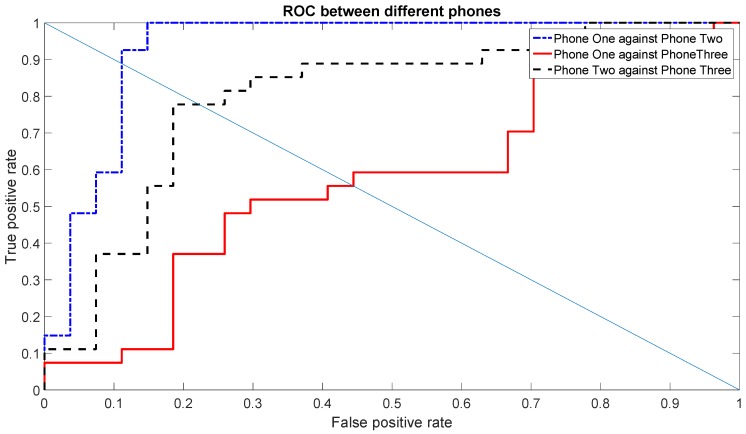
ROCs among different phones using the first scenario for Gyroscope X for a single day’s data in the second set of measurements.

**Figure 13:**
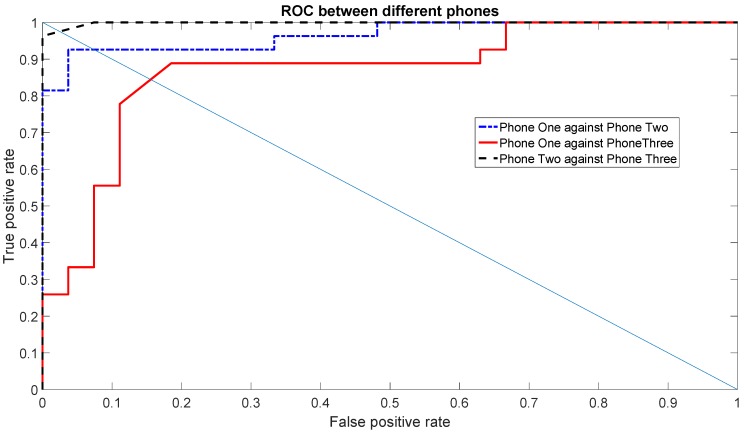
ROCs among different phones using the first scenario for Gyroscope Y for a single day’s data in the second set of measurements.

This was also confirmed by the ROC curves and the related EER values, which are calculated on the measurement session executed four months after the first session. The ROC curves are calculated with the same parameters provided above (Gyroscope X for the set of features (1, 2, 3, 5, 7) and Gyroscope Y for the set of features (1, 3, 5, 6, 7)), and they are shown in Figure 12 and Figure 13.

As pointed out before, the EER values from the second set of measurements (provided in Table 5), show that the identification accuracy using the gyroscope X is worse than the Gyroscope Y. This is also due to the relative low accuracy in distinguishing Phone One against Phone Three, which was already shown in the previous graphs (e.g., Figure 6). Regardless of the overall accuracy, we note that the EER values with the Gyroscope Y are also quite consistent and very similar between the first set of measurements (*i.e.*, Table 4) and the second set of measurements (*i.e.*, Table 5). As a consequence, we recommend the use of Gyroscope Y for the results stability as well.

This is the end of the analysis presented in this paper, which had the objective to identify the best combination of features and MEMS components to improve the accuracy in smartphone identification. We started this analysis with the evaluation of the different statistical features and different kernel functions using the Accelerometer in the *X* direction. Even if the resulting accuracy was good (around 70% in most cases), we tried to identify the optimum values to increase this accuracy. This was obtained by combining different features, and we have exhaustively evaluated all of the possible combinations of statistical features. Finally, we have evaluated the other components of the built-in MEMS (e.g., Gyroscope), and we have achieved the high level of accuracy presented in this section for specific combination of features. We have executed another set of measurements four months after the first set of measurements to show that the algorithm is generalizable. Even if the classification using the Gyroscope X was slightly degraded, the classification using Gyroscope Y maintained almost the same level of accuracy. The resulting level of accuracy is suitable for many of the applications described in the introduction. Obviously, these results are obtained on the basis of a specific scenario and the choice of the parameters may be slightly different in a different scenario. Still, we think that the chosen scenario has a good balance between the need for a simple configuration of the robotic arm and the need for an adequate stimulation of the MEMS components in the smartphone.

## 5. Conclusions

In this paper, we have investigated the problem of identifying phones on the basis of their MEMS components like Accelerometers and Gyroscopes. We have used various statistical features with different values of the parameters and thresholds. The main objective of the study was to identify which statistical features provide the better accuracy results. To achieve this objective, we have conducted an exhaustive analysis on the selection and combination of statistical features with different parameters and different kernel functions of the Support Vector Machine (SVM) used for binary classification. To verify the robustness of the algorithm, we have verified the resulting accuracy with data taken from four different stimulating scenarios in different sessions separated in time by four months. The results have shown that it is possible to obtain very high accuracy to distinguish different smartphones. In comparison to previous work, we have also identified the statistical entropy features as very important features to improve the identification accuracy. This is a relevant result, as previous work did not use statistical entropy features or used them in a limited way. As a consequence, the digital output of the MEMSs of a smartphone submitted to a specific repeatable scenario can be used to identify different smartphones of the same model, which can be used both for fighting against counterfeiting or for security and forensics purposes. Future work will investigate the impact on identification accuracy of different platforms for the generation of motion patterns, and it will enlarge the number of mobile phones of the same brand and model to be used in the experiment.

## Figures and Tables

**Table 1:** Optimum values of the threshold parameter for the Entropy statistical features for each axis of the Accelerometer and Gyroscope.

MEMS Component	Threshold Entropy	Sure Entropy	Norm Entropy
Accelerometer X	1.1	1.1	2.5
Accelerometer Y	2.0	2.0	2.6
Accelerometer Z	1.8	1.9	1.1
Gyroscope X	0.1	0.1	2.7
Gyroscope Y	0.1	0.8	2.5
Gyroscope Z	0.1	0.1	3.0

**Table 2:** Results of the second round of measurements four months after the initial ones. Data for Gyroscope X.

Set of Features	Indirect Comparison	Direct Comparison
[2 5 6 7 8]	62.65	80.24
[1 2 3 5 7]	58.8	76.71
[2 5 6 7 8]	58.33	79.5988

**Table 3:** Results of the second round of measurements four months after the initial ones. Data for Gyroscope Y.

Set of Features	Indirect Comparison	Direct Comparison
[1 3 5 6 7]	58.33	91.09
[2 3 4 5 6]	54.32	83.95
[3 5 6 7 8]	57.09	90.43

**Table 4:** Equal Error Rate (EER) for Gyroscope X and Gyroscope Y for a single day’s data in the first set of measurements campaigns.

Equal Error Rate (EER)	Gyroscope X	Gyroscope Y
Phone One against Phone Two	0.15	0.04
Phone One against Phone Three	0.12	0.17
Phone Two against Phone Three	0.12	0.07

**Table 5:** EER for Gyroscope X and Gyroscope Y for a single day’s data in the second set of measurements campaigns.

EER	Gyroscope X	Gyroscope Y
Phone One against Phone Two	0.11	0.04
Phone One against Phone Three	0.44	0.16
Phone Two against Phone Three	0.22	0.07

## Supplementary Materials

The following are available online at http://www.mdpi.com/1424-8220/16/6/818/s1, Data D1: First set of sensor data in 2015, Data D2: Second set of sensor data in 2016.

## Abbreviations

The following abbreviations are used in this manuscript:

EER: Equal Error Rate
IC: Integrated Circuit
KKT: Karush–Kuhn–Tucker
MEMS: Micro Electro-Mechanical Systems
MLP: Multilayer Perceptron
PKI: Public Key Infrastructure
RBF: Radial Basis Function
RF: Radio Frequency
ROC: Receiver Operating Characteristics
SMO: Sequential Minimal Optimization
SVM: Support Vector Machine
